# (Pro)renin receptor accelerates development of sarcopenia *via* activation of Wnt/YAP signaling axis

**DOI:** 10.1111/acel.12991

**Published:** 2019-07-08

**Authors:** Naohiro Yoshida, Jin Endo, Kenichiro Kinouchi, Hiroki Kitakata, Hidenori Moriyama, Masaharu Kataoka, Tsunehisa Yamamoto, Kohsuke Shirakawa, Satoshi Morimoto, Akira Nishiyama, Akihiro Hashiguchi, Itsuro Higuchi, Keiichi Fukuda, Atsuhiro Ichihara, Motoaki Sano

**Affiliations:** ^1^ Department of Endocrinology and Hypertension Tokyo Women’s Medical University Tokyo Japan; ^2^ Department of Cardiology, School of Medicine Keio University Tokyo Japan; ^3^ Department of Internal Medicine, School of Medicine Keio University Tokyo Japan; ^4^ Department of Pharmacology, Faculty of Medicine Kagawa University Kagawa Japan; ^5^ Department of Neurology and Geriatrics, Graduate School of Medical and Dental Sciences Kagoshima University Kagoshima Japan; ^6^ School of Health Sciences, Faculty of Medicine Kagoshima University Kagoshima Japan

**Keywords:** (pro)renin receptor, aging, canonical Wnt pathway, sarcopenia, skeletal muscle atrophy, YAP signaling

## Abstract

To extend life expectancy and ensure healthy aging, it is crucial to prevent and minimize age‐induced skeletal muscle atrophy, also known as sarcopenia. However, the disease's molecular mechanism remains unclear. The age‐related Wnt/β‐catenin signaling pathway has been recently shown to be activated by the (pro)renin receptor ((P)RR). We report here that (P)RR expression was increased in the atrophied skeletal muscles of aged mice and humans. Therefore, we developed a gain‐of‐function model of age‐related sarcopenia *via* transgenic expression of (P)RR under control of the CAG promoter. Consistent with our hypothesis, (P)RR‐Tg mice died early and exhibited muscle atrophy with histological features of sarcopenia. Moreover, Wnt/β‐catenin signaling was activated and the regenerative capacity of muscle progenitor cells after cardiotoxin injury was impaired due to cell fusion failure in (P)RR‐Tg mice. In vitro forced expression of (P)RR protein in C2C12 myoblast cells suppressed myotube formation by activating Wnt/β‐catenin signaling. Administration of Dickkopf‐related protein 1, an inhibitor of Wnt/β‐catenin signaling, and anti‐(P)RR neutralizing antibody, which inhibits binding of (P)RR to the Wnt receptor, significantly improved sarcopenia in (P)RR‐Tg mice. Furthermore, the use of anti‐(P)RR neutralizing antibodies significantly improved the regenerative ability of skeletal muscle in aged mice. Finally, we show that Yes‐associated protein (YAP) signaling, which is coordinately regulated by Wnt/β‐catenin, contributed to the development of (P)RR‐induced sarcopenia. The present study demonstrates the use of (P)RR‐Tg mice as a novel sarcopenia model, and shows that (P)RR‐Wnt‐YAP signaling plays a pivotal role in the pathogenesis of this disease.

## 1 INTRODUCTION

Developed countries are characterized by a rapidly aging population. Age‐related muscle atrophy, also known as sarcopenia, is associated with adverse health outcomes including disability, hospitalization, poor quality of life, and mortality. Treatment and prevention of sarcopenia are important for extending both healthy life expectancy and longevity. Until recently, a progressive loss of muscle mass and muscle strength in the elderly was considered as an inevitable sign of physical aging. Several mechanisms underlying sarcopenia have been proposed, including impairment of muscle regenerative capacity, imbalance between muscle protein synthesis and degradation, and chronic inflammation (Arnold, Egger, & Handschin, 2011). Importantly, they have become the focus of various treatments.

The Wnt/β‐catenin signaling pathway plays important roles in embryogenesis, carcinogenesis, and self‐renewal/differentiation of stem cells (Moon, Kohn, De Ferrari, & Kaykas, 2004). It is also known to promote the aging phenotype (Naito et al., 2012) and is associated with the decreased regenerative ability of muscle progenitor cells (Brack et al., 2007). (Pro)renin receptor ((P)RR) is an activator of Wnt/β‐catenin signaling (Cruciat et al., 2010). (P)RR enhances angiotensin I production by increasing the catalytic activity of renin and prorenin (Nguyen et al., 2002). It also functions as a subcomponent of vacuolar H^+^‐ATPase (V‐ATPase), which is responsible for lysosomal acidification (Kinouchi et al., 2010). Moreover, it has been recently reported to activate Wnt/β‐catenin signaling by acting as an adaptor between the Wnt receptor and V‐ATPase (Cruciat et al., 2010). However, it remains largely unknown whether (P)RR mediates the activation of Wnt/β‐catenin signaling in aging and if so, how (P)RR is involved in the pathogenesis of senescence‐related muscle atrophy.

The Hippo/Yes‐associated protein (YAP) signaling pathway that regulates organ growth in mammals is associated with differentiation and proliferation of muscle cells (Zhao, Tumaneng, & Guan, 2011). Constitutive activation of YAP signaling in skeletal muscles was shown to result in muscle degeneration and atrophy (Judson et al., 2013; Watt et al., 2015). A recent study showed that YAP regulated Wnt signaling through its release from the β‐catenin destruction complex to promote the transcription of its target genes (Azzolin et al., 2014). However, little is known about the role of YAP signaling in skeletal muscle aging.

In this study, we sought to investigate how (P)RR activation of the Wnt pathway affected signal transduction and cellular function in the pathogenesis of sarcopenia. We demonstrated an increase in (P)RR protein expression following activation of the Wnt pathway in atrophied skeletal muscles of senile mice and humans. The (P)RR‐transgenic (Tg) mice generated in this study exhibited unique skeletal muscle atrophy with features of sarcopenia. Forced expression of (P)RR in skeletal muscles activated both the Wnt/β‐catenin and YAP signaling pathways, resulting in disturbed skeletal muscle growth *via* impairment of myoblast fusion during regeneration following muscle injury. Inhibition of (P)RR‐Wnt‐YAP signaling improved senile muscle atrophy, suggesting that such blocking agents are promising therapeutic candidates against sarcopenia.

## 2 RESULTS

### 2.1 (P)RR‐TG MICE EXHIBIT SKELETAL MUSCLE ATROPHY

**Figure 1:**
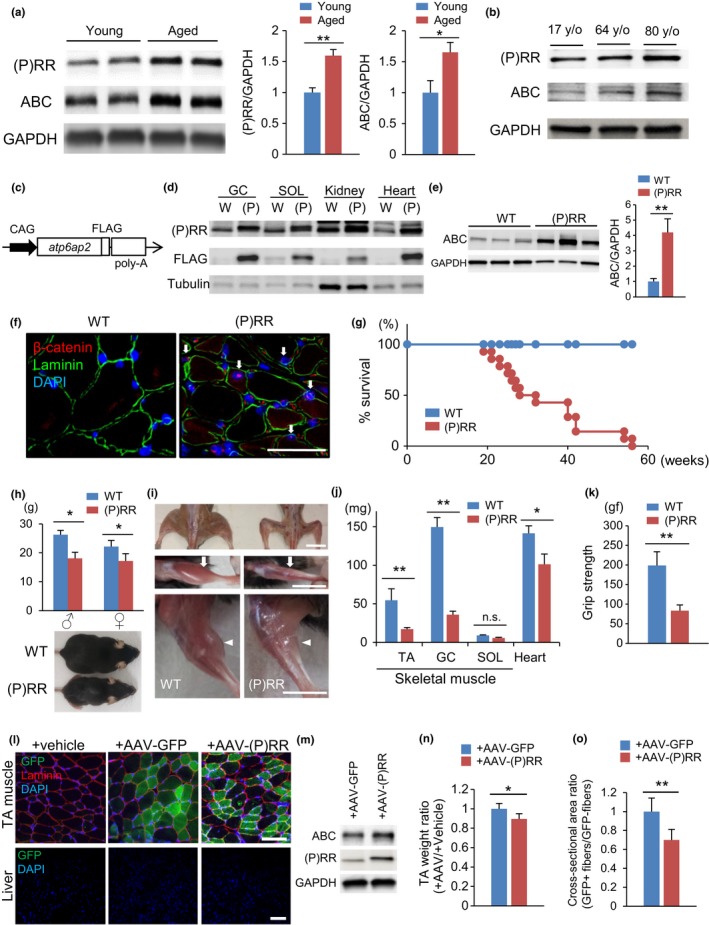
(P)RR‐Tg mice exhibit skeletal muscle atrophy with activation of β‐catenin signaling. (a) Western blotting of (pro)renin receptor ((P)RR) and active β‐catenin (ABC) expression in total protein extracts from the gastrocnemius muscle (GC) of young (12‐week‐old) and aged 100‐week‐old) WT mice (left) and quantification by densitometry (right) (*n* = 6). (b) (P)RR and ABC protein levels in total protein extracts from human muscles of 17‐, 64‐, and 80‐year‐old volunteers (<40 y/o: *n* = 3; 40–60 y/o: *n* = 3; 65 y/o<: *n* = 3). (c) Schematic representation of the transgenic construct. FLAG‐tagged murine *atp6ap2* ((P)RR) cDNA is expressed under control of the CAG promoter. (d) Western blotting of forced (P)RR and FLAG protein expression in GC, SOL, kidney, and heart from (P)RR‐Tg mice. (e) Western blotting of ABC expression in total protein extracts from the GC of WT and (P)RR‐Tg mice (left) and quantification by densitometry (right) (*n* = 6). (f) Immunohistochemical staining with anti‐ABC (red) and anti‐laminin (green) antibodies showing translocation of β‐catenin into the nucleus of GC cells in (P)RR‐Tg mice. Nuclei were labeled with DAPI (blue). Scale bar, 100 mm. (g) Kaplan–Meier survival curve for WT and (P)RR‐Tg mice (*n* = 12). (h) Body weight in WT and (P)RR‐Tg mice. The data of female or male (P)RR‐Tg mice are presented relative to their same‐sex WT counterparts (*n* = 8) (top). Whole‐body image of WT and (P)RR‐Tg mice (bottom). Scale bar, 10 mm. (i) Muscle atrophy in TA (white arrow) and GC (white arrowhead) of (P)RR‐Tg mice. (j) Comparison between muscle (TA, GC, and SOL) and heart weight between WT and (P)RR‐Tg mice (*n* = 7). (k) Handgrip strength of WT and (P)RR‐Tg mice (*n* = 8). (l) Immunostaining for GFP (green) and laminin (red) in cross sections of AAV‐GFP‐injected or AAV‐(P)RR‐injected TA and vehicle‐injected contralateral TA in 8‐week‐old WT mice. Nuclei were labeled with DAPI (blue). Scale bar, 100 mm. (m) Western blotting of ABC and (P)RR in total protein extracts from TA injected with AAV‐(P)RR or AAV‐GFP. (n) Comparison of the weight ratio of AAV‐treated versus vehicle‐treated TA (*n* = 5). (o) Comparison of cross‐sectional area ratio of GFP‐positive to GFP‐negative myofibers in AAV‐treated TA (*n* = 5, *N* = 100 per group). Data represent the mean ± *SEM*. *p < 0.05 and **p < 0.01; n.s., not significant, as determined by the Mann–Whitney *U* test

To assess whether (P)RR expression increased coordinately with the activation of Wnt/β‐catenin signaling in skeletal muscles, we compared (P)RR and nonphosphorylated β‐catenin (active‐β‐catenin [ABC]) protein levels in skeletal muscles in young and senile mice. The expression of (P)RR and ABC protein was higher in the skeletal muscles of senile mice than in those of young mice (Figure 1a). Similarly, ABC and (P)RR protein levels increased gradually with aging in the skeletal muscles of healthy human volunteers (Figure 1b).

To elucidate whether the increase in (P)RR expression affected muscle mass and strength, we generated (P)RR‐Tg mice by expressing FLAG‐tagged mouse ATP6AP2/(P)RR under control of the CAG promoter (Figure 1c). (P)RR‐Tg mice produced the transgene protein abundantly in the whole body, particularly in the heart, skeletal muscles, kidneys, and liver (Figure 1d). As expected, ABC was significantly increased (Figure 1e) and nuclear localization of β‐catenin could be observed in the muscles of (P)RR‐Tg mice, indicating activation of Wnt/β‐catenin signaling by (P)RR (Figure 1f).

(P)RR‐Tg mice were born in Mendelian frequencies; however, all of them were dead within 1 year after birth (Figure 1g). Consistent with our hypothesis, (P)RR‐Tg mice exhibited small body weight and reduced muscle mass without impaired bone formation (Figures 1h,I and S1A,B). Skeletal muscle atrophy in (P)RR‐Tg mice was selectively pronounced in fast muscles, such as the gastrocnemius muscle (GC) and tibialis anterior muscle (TA), but not in slow muscles such as the soleus muscle (SOL) (Figure 1j). Moreover, the grip strength of (P)RR‐Tg mice was significantly lower than that of WT mice (Figure 1k). The diaphragm muscle, an inspiratory muscle composed of an equal proportion of fast and slow fibers, was also strikingly atrophied in (P)RR‐Tg mice. This finding suggests that progressive respiratory failure by the atrophied diaphragm muscle might lead to the premature death seen in (P)RR‐Tg mice (Figure S1C). Although the transgene protein was strongly expressed in the liver and kidney of (P)RR‐Tg mice, these organs’ functions were within normal according to blood tests (Figure S1D,E) Interestingly, β‐catenin was activated only in fast muscles, but not in other organs, even in the presence of increased (P)RR expression (Figure S1F). Notably, the heart of (P)RR‐Tg mice strongly expressed (P)RR with no concomitant activation of β‐catenin, cardiac dysfunction, fibrosis, or macrophage accumulation (Figure S1G‐J).

To determine whether expression of (P)RR in skeletal muscle directly induced muscle atrophy, we injected the adeno‐associated virus (AAV) vector into the TA of 8‐week‐old mice using a thermo‐reversible gelation polymer. This strategy induced strong expression of (P)RR only in the tibial muscle of the AAV‐injected limb, but not in the muscle of the noninjected contralateral limb and liver (Figure 1l). ABC expression was higher in the AAV‐(P)RR‐injected limb than in the control vector‐injected limb (Figure 1m). Moreover, weight and fiber size were significantly lower in the muscle in which (P)RR was highly expressed by AAV compared to control muscles injected with the AAV‐GFP vector (Figure 1n,o).

Further, we assessed whether vasculature or motor neurons were involved in muscle atrophy. Vascular staining with lectin revealed no major difference in vasculature density in the muscle of (P)RR‐Tg and WT mice (Figure S1K). As the amount of (P)RR did not change between healthy muscles and neurogenic muscles atrophied by denervation (Figure S1L), we concluded that the vasculature and motor neurons had only a minor effect on (P)RR‐related muscle atrophy.

### 2.2 Skeletal muscle atrophy observed in (P)RR‐Tg mice is consistent with the aging phenotype

**Figure 2:**
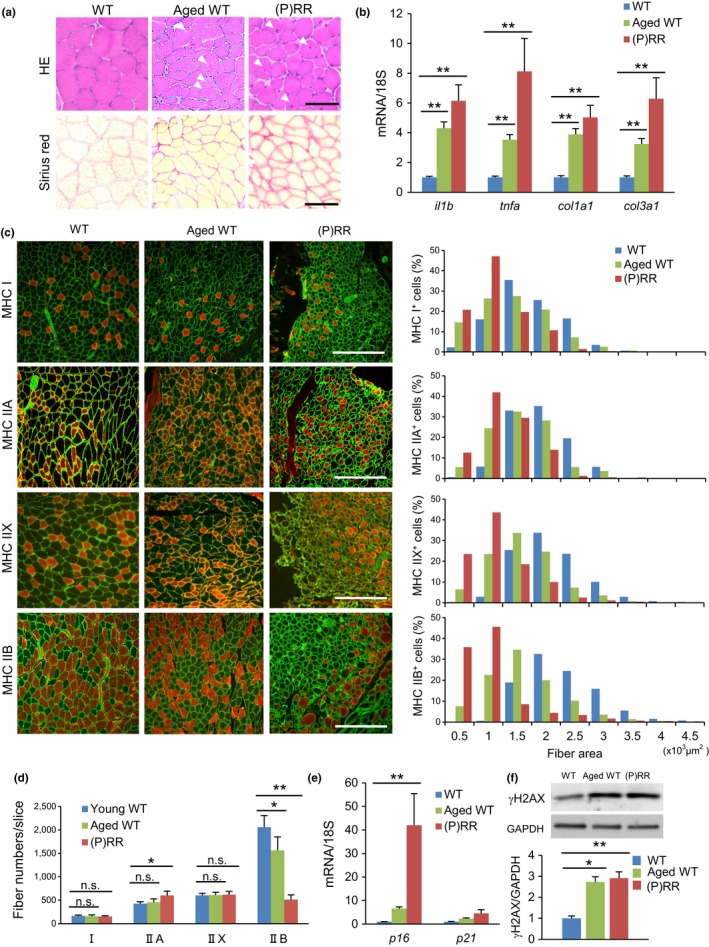
(P)RR‐Tg mice exhibit a sarcopenia‐like phenotype. (a) Histological sections with hematoxylin and eosin staining (top) and sirius red staining (bottom) of the gastrocnemius muscle (GC) in WT (12‐week‐old), aged WT (100‐week‐old), and (P)RR‐Tg mice. White arrowheads indicate central nuclei during ongoing myofiber repair. Scale bars, 100 mm. (b) Relative expression levels of inflammation and fibrosis‐related genes (*il1b, tnfa, col1a1, col3a1*) in GC of WT, aged WT, and (P)RR‐Tg mice. Expression levels were normalized to those of 18S ribosomal RNA and then to those in the GC of WT mice (*n* = 7). (c) Immunofluorescence staining for MHC Ⅰ, IIA, and IIB (red) and laminin (green) in cross sections of TA in WT, aged WT, and (P)RR‐Tg mice. Nuclei were labeled with DAPI (blue). Scale bars, 300 mm (left). Cross‐sectional area of individual myofibers positive for each MHC isoform (I, IIA, and IIB) in the GC of WT, aged WT, and (P)RR‐Tg mice (*n* = 6, *N* = 450–500 per group) (right). (d) Number of muscle fibers positive for MHC I, IIA, and IIB per slice (*n* = 7). (e) Relative expression levels of senescence marker genes (*p16* and *p21*) in GC of WT, aged WT, and (P)RR‐Tg mice. Expression levels were normalized to those of 18S ribosomal RNA and then to those in the GC of WT mice (*n* = 6). (f) Western blotting of γH2AX expression in total protein extracts from GC of WT, aged WT, and (P)RR‐Tg mice (top) and quantification by densitometry (bottom). GAPDH was used as internal control for total protein (*n* = 6). Data represent the mean ± *SEM*. *p < 0.05 and **p < 0.01; n.s., not significant, as determined by the Mann–Whitney *U* test (b,e,f) or ANOVA followed by the Bonferroni post hoc correction (d)

The skeletal muscle fibers of the (P)RR‐Tg mouse became smaller and rounder than those of the WT littermate (Figure 2a). Sirius red staining revealed that interstitial fibrotic tissue increased in (P)RR‐Tg mice to the same extent as seen in aged WT mice (Figure 2a). Interestingly, despite breeding in the absence of stress conditions, newly regenerated muscle fibers with central nuclei, a common feature of aging muscle, were sporadically observed in (P)RR‐Tg mice (Figure 2a). In addition, immunostaining revealed that inflammatory cells expressing CD68, a macrophage marker, were recruited and accumulated in the fibrotic area in the muscles of the (P)RR‐Tg mouse (Figure S2A). As in aged WT mice, mRNA expression of tumor necrosis factor (TNF)‐ α, interleukin (IL)‐1β, and collagen types I and III was increased in (P)RR‐Tg mice (Figure 2b). Surprisingly, electron microscopic analysis revealed only narrowing of muscle filaments, but no muscle fiber misalignment, no mitochondrial structural abnormality, and no increase in autophagosomes in (P)RR‐Tg mice (Figure S2B).

Glycolytic type II muscle fibers are bigger and are preferentially lost, whereas type I muscle fibers are commonly preserved in age‐related muscle atrophy (Lexell & Downham, 1992). To determine which type of muscle fiber preferentially decreased and atrophied in (P)RR‐Tg mice, myosin heavy chain (MHC) type I (slow filament), type IIA (intermittent), type IIX, and type IIB (fast filament) were immunostained, and the area and number of each myofiber were measured in muscle cross sections. The cross‐sectional area (CSA) of every fiber type was smaller in (P)RR‐Tg mice and aged WT mice than in young WT mice. The number of MHC IIB‐positive myofibers dramatically decreased in (P)RR‐Tg mice, whereas the number of MHC I‐ and MHC IIX‐positive myofibers did not differ (Figure 2c,d). Moreover, aging markers, such as *p16*, *p21*, and γH2AX, were upregulated in the muscles of both (P)RR‐Tg mice and aged WT mice (Figure 2e,f). Taken together, these findings indicated that (P)RR‐Tg mice exhibited a sarcopenia‐like phenotype.

### 2.3 Autophagy dysfunction is an initial trigger of sarcopenia in (P)RR‐Tg mice

Muscle atrophy occurs when protein degradation exceeds protein synthesis. The ubiquitin–proteasome pathway (UPP) is a major protein degradation system, whereas mTOR is a crucial player in protein synthesis (Bodine et al., 2001). We examined whether UPP and mTOR pathways were involved in muscle atrophy in (P)RR‐Tg mice. Unexpectedly, muscle atrophy‐related UPP genes, including muscle atrophy F‐box (MAFbx)/atrogin‐1 (*fbxo32*), muscle RING finger 1 (MuRF1; *trim63*), and myostatin (*mstn*), were downregulated (Figure S3A). In contrast, mTOR and its downstream targets, including ribosomal protein S6 kinase 1 (S6K1; *rps6kb1*) and eukaryotic translation initiation factor 4E‐binding protein 1 (4E‐BP1; *eif4ebp1*), were activated in (P)RR‐Tg mice (Figure S3B). These results indicated that UPP was suppressed and the mTOR pathway was activated in (P)RR‐Tg mice, resulting in strong protein synthesis. These actions could be aimed at compensating for muscle that was atrophied and lost due to another cause. The autophagy–lysosome pathway is another major protein degradation pathway related to muscle atrophy (Sandri, 2010). (P)RR‐Tg mice exhibited accumulation of the ubiquitin‐associated protein p62, a reliable marker of autophagy, and a defect in microtubule‐associated protein light chain 3 (LC3) conversion from LC3‐I to LC3‐II, a gold standard for autophagosome formation. However, no alteration in lysosomal‐associated membrane protein 2A, an established component of chaperone‐mediated autophagy, was observed. These findings indicated that muscle atrophy in (P)RR‐Tg mice was characterized by autophagic dysfunction (Figure S3C,D).

To determine whether autophagy dysfunction was an initial trigger of sarcopenia in (P)RR‐Tg mice, we analyzed muscle tissue of 3‐ and 5‐week‐old mice. Until 3 weeks of age, there was no obvious histological difference between (P)RR‐Tg mice and their control littermates (Figure S3E). From about 5 weeks after birth, muscle fibers with a central nucleus showing regeneration began to emerge in (P)RR‐Tg mice, together with muscle atrophy (Figure S3E). An increase in LC3 and its defective conversion could be detected already at 3 weeks of age, in line with activation of Wnt signaling (Figure S3F). Additionally, TUNEL‐positive apoptotic cells, which tend to increase in the muscle with age, were observed in 5‐week‐old (P)RR‐Tg mice (Figure S3G,H). Taken together, these results suggested that autophagy dysfunction induced by the activation of (P)RR‐Wnt‐YAP signaling caused initiation of sarcopenia in (P)RR‐Tg mice and led to myocytes’ death and subsequent muscle regeneration.

Next, we investigated whether (P)RR functions other than Wnt signaling activation affected muscle atrophy. In the muscles of (P)RR‐Tg mice, the levels of prorenin and renin, which can be ligands for (P)RR, were not elevated, whereas the concentration of angiotensin II, which is produced locally by the enzymatic activity of (P)RR, was very small and comparable to that of WT mice (Figure S3I,J). Similarly, the extracellular signal‐regulated kinase (ERK) signaling pathway, which could be activated by (P)RR independently of its renin activity (Huang et al., 2006), appeared unchanged (Figure S3K). Finally, even the lysosomal pH of myocytes expressing (P)RR was within normal, indicating no impact as a subcomponent of V‐ATPase (Figure S3L).

### 2.4 Muscle regeneration after CTX injection is impaired due to altered muscle progenitor cell fusion in (P)RR‐Tg mice

**Figure 3:**
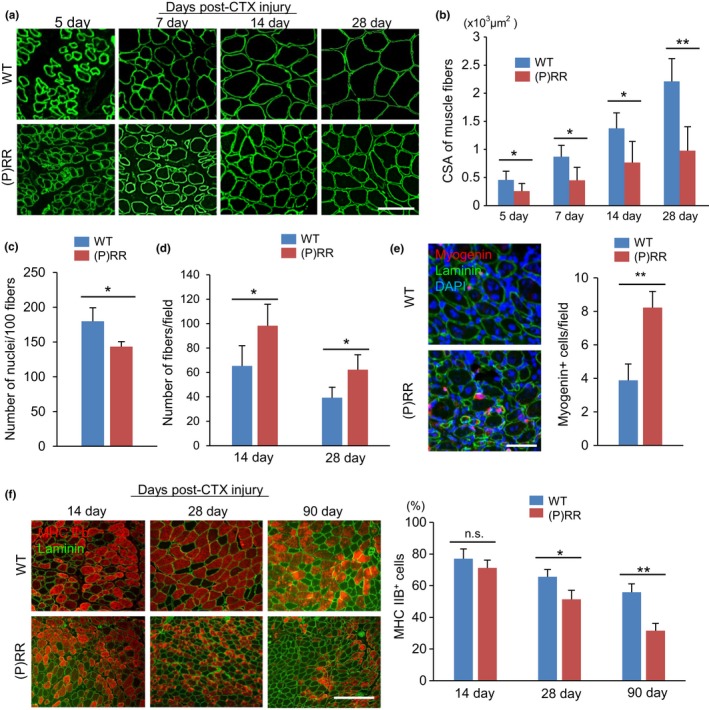
Histological recovery of skeletal muscle after cardiotoxin (CTX) injection is impaired in (P)RR‐Tg mice. (a) Immunostaining for laminin (green) in cross sections of TA in WT and (P)RR‐Tg mice on day 5, 7, 14, and 28 after CTX injection. Scale bars, 100 mm. (b) Cross‐sectional area of regenerated muscle fibers in WT and (P)RR‐Tg mice on days 5, 7, 14, and 28 after CTX injury (*n* = 6). (c) Number of nuclei per myofiber in WT and (P)RR‐Tg mice on day 14 after CTX injection (*n* = 6). (d) Number of central nuclear myocytes per cross section in WT and (P)RR‐Tg mice on days 14 and 28 after CTX injection (*n* = 6). (e) Immunofluorescent staining for myogenin (red) and laminin (green) in cross sections of TA in WT and (P)RR‐Tg mice on day 7 after CTX injection. Nuclei were labeled with DAPI (blue) (left). Numbers of myogenin‐positive regenerating myocytes in WT and (P)RR‐Tg mice on day 7 after CTX injection (right) (*n* = 6). Scale bars, 100 mm. (f) Immunofluorescence staining for MHC IIB (red) and laminin (green) in cross sections of TA in WT and (P)RR‐Tg mice on days 14, 28, and 90 after CTX injection (left). Scale bars, 300 mm. Percentage of MHC IIB‐positive myocytes in cross sections of TA after CTX injection (right) (*n* = 6). Data represent the mean ± *SEM*. *p < 0.05 and **p < 0.01; n.s., not significant, as determined by the Mann–Whitney *U* test

One proposed mechanism underlying age‐related muscle atrophy is the decline in myogenic capacity of myoblasts. To assess the regenerative potential of muscle progenitor cells, we induced skeletal muscle injury by injecting cardiotoxin (CTX) into the TA muscle and compared the regenerative process histologically. During muscle regeneration, in the 28 days following CTX injection, we measured the CSA and number of regenerated muscle fibers in WT and (P)RR‐Tg mice. The muscle fibers tended to grow in both types of mice; however, the rate was much slower in (P)RR‐Tg mice than in WT animals (Figure 3a,b). Moreover, multinucleated fused myocytes were frequently observed in WT mice, whereas they were rarely present in (P)RR‐Tg mice (Figure 3c). The number of muscle fibers was constantly higher in (P)RR‐Tg mice than in WT mice until 28 days after CTX injection (Figure 3d), suggesting that the decrease in myogenic capacity in (P)RR‐Tg mice was a result of impaired myoblast fusion. A recent study showed that conditional activation of Wnt/β‐catenin in vitro and in vivo augmented the precocious differentiation of muscle progenitor cells, as indicated by high expression of myogenin, a marker of myoblast differentiation (Rudolf et al., 2016). The number of myogenin‐expressing regenerated cells on day 7 after CTX injection was higher in (P)RR‐Tg mice than in WT mice (Figure 3e). Until day 14 post‐CTX injection, almost all regenerated myocytes expressed MHC IIB even in (P)RR‐Tg mice; however, the percentage of MHC IIB‐positive fibers in (P)RR‐Tg mice gradually decreased and 90 days later reached almost the same level as before administration (Figure 3f).

### 2.5 Myogenic differentiation is disturbed by the activation of Wnt/β‐catenin signaling in (P)RR‐expressing C2C12 myoblast cells

**Figure 4:**
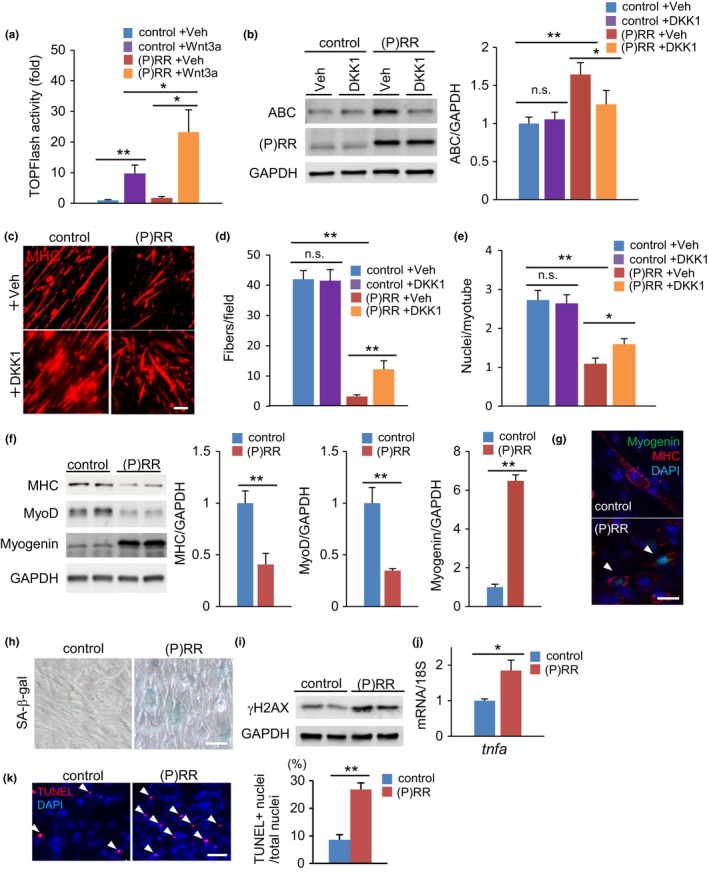
Myotube formation in (pro)renin receptor ((P)RR)‐expressing C2C12 myoblasts is impaired by cell fusion failure due to activation of (P)RR‐induced Wnt signaling. (a) TOPFlash activity of control and (P)RR‐expressing C2C12 myoblasts with or without Wnt3a stimulation (*n* = 7). (b) Western blotting of active β‐catenin (ABC) and (P)RR expression in total protein extracts from a C2C12 cell line stably expressing (P)RR cultured in growth medium with vehicle or DKK1 (left) and quantification by densitometry (right) (*n* = 6). GAPDH was used as internal control for total protein. (c) Immunofluorescence analysis with anti‐MHC antibody of control and (P)RR‐expressing C2C12 cells cultured in differentiation medium to induce myotube formation. Scale bars, 100 mm. (d) Number of MHC‐positive myotubes per field of high‐magnified view (×400) in control and (P)RR‐expressing C2C12 myoblasts after 4 days in differentiation medium, with or without DKK1 treatment (*n* = 6). (e) Number of nuclei per myotube formed in control and (P)RR‐expressing C2C12 cells with or without DKK1 treatment (*n* = 6). (f) Western blotting of MHC, MyoD, and myogenin expression in total protein extracts from a C2C12 cell line stably expressing (P)RR cultured in differentiated medium (left) and quantification by densitometry (*n* = 6) (right). GAPDH was used as internal control for total protein. (g) Immunostaining for myogenin (green) and laminin (red) in control and (P)RR‐expressing C2C12 myotubes. Nuclei were labeled with DAPI (blue). Scale bar, 50 mm. (h) SA‐β‐gal staining in control and (P)RR‐expressing C2C12 myotubes. Scale bars, 50 mm. (i) Western blotting of γH2AX in total protein extracts of control and (P)RR‐expressing C2C12 myotubes. (j) Relative expression levels of the *tnfa* gene in control and (P)RR‐expressing C2C12 myotubes (*n* = 6). (k) TUNEL staining (left) and the ratio of TUNEL‐positive nuclei to total nuclei in control and (P)RR‐expressing C2C12 myotubes. (*n* = 5, *N* = 100 per group) Scale bars, 50 mm. Data represent the mean ± *SEM*. *p < 0.05 and **p < 0.01; n.s., not significant, as determined by the Mann–Whitney *U* test (f,j,k) or ANOVA followed by the Bonferroni post‐hoc correction (a,b,d,e)

To examine whether (P)RR protein impacted the ability of myoblasts to differentiate into myotubes via activation of Wnt signaling, we generated C2C12 myoblast cells stably expressing (P)RR by using a retroviral vector. The TOPFlash luciferase assay revealed higher Wnt/T‐cell factor (TCF)‐driven transcription in (P)RR‐expressing myoblasts than in control cells, either in the absence or in the presence of Wnt3a stimulation (Figure 4a). As with the atrophied muscles of (P)RR‐Tg mice, active β‐catenin (ABC) expression was high in the myotubes derived from (P)RR‐expressing myoblasts. Upregulation of (P)RR expression strikingly impaired the efficiency of myotube formation and multinucleation in C2C12 myoblast cells; however, Dickkopf‐related protein 1 (DKK1), a Wnt inhibitor, blocked β‐catenin activation and restored myotube function (Figure 4b–e). In vitro assessment of myogenic markers in (P)RR‐expressing C2C12 cells revealed significantly increased myogenin expression but decreased myoD expression, confirming in vivo results of a precocious differentiation in (P)RR‐expressing myocytes (Figure 4f,g). In the differentiated myotube from (P)RR‐expressing C2C12 cells, we detected positive SA‐β‐gal staining with an increase in γH2AX, a senescent marker, and increased expression of TNF‐α, a senescence‐associated secretory phenotype, indicating that (P)RR expression induced cellular senescence (Figure 4h‐j). Further, apoptotic cells were frequently observed among (P)RR‐expressing cells after induction of differentiation (Figure 4k).

### 2.6 Treatment with DKK1, an inhibitor of Wnt/β‐catenin signaling, attenuates muscle atrophy in (P)RR‐Tg mice

Next, to determine whether activation of Wnt/β‐catenin signaling contributed to (P)RR‐induced muscle atrophy, we injected DKK1 into the TA muscle of (P)RR‐Tg mice and evaluated improvement in muscle atrophy following inhibition of Wnt signaling. Five days after DKK1 injection, β‐catenin activation in the muscles of (P)RR‐Tg mice was blocked (Figure S4A), and the atrophied muscles in (P)RR‐Tg mice became significantly thicker, while normal muscles in control littermates showed no change (Figure S4B). DKK1 treatment enlarged the CSA of myofibers only in (P)RR‐Tg mice (Figure S4C,D). The expression of genes associated with fibrosis, inflammation, and aging, which were upregulated in (P)RR‐Tg mice and aged WT mice, was suppressed after DKK1 treatment (Figure S4E,G). Additionally, myogenin was immunostained in the many nuclei of the muscle fiber in (P)RR‐Tg mice even under unstressed conditions, and administration of DKK1 significantly attenuated the number of myogenin‐positive fibers (Figure S4F). As above, pharmacological inhibition of Wnt/β‐catenin signaling could improve muscle atrophy in (P)RR‐Tg mice, indicating that the activation of Wnt signaling was responsible for the development of (P)RR‐induced muscle atrophy.

### 2.7 Administration of anti‐(P)RR neutralizing antibody to inhibit (P)RR‐induced Wnt signaling activation attenuates muscle atrophy in (P)RR‐Tg mice and senile WT mice

**Figure 5:**
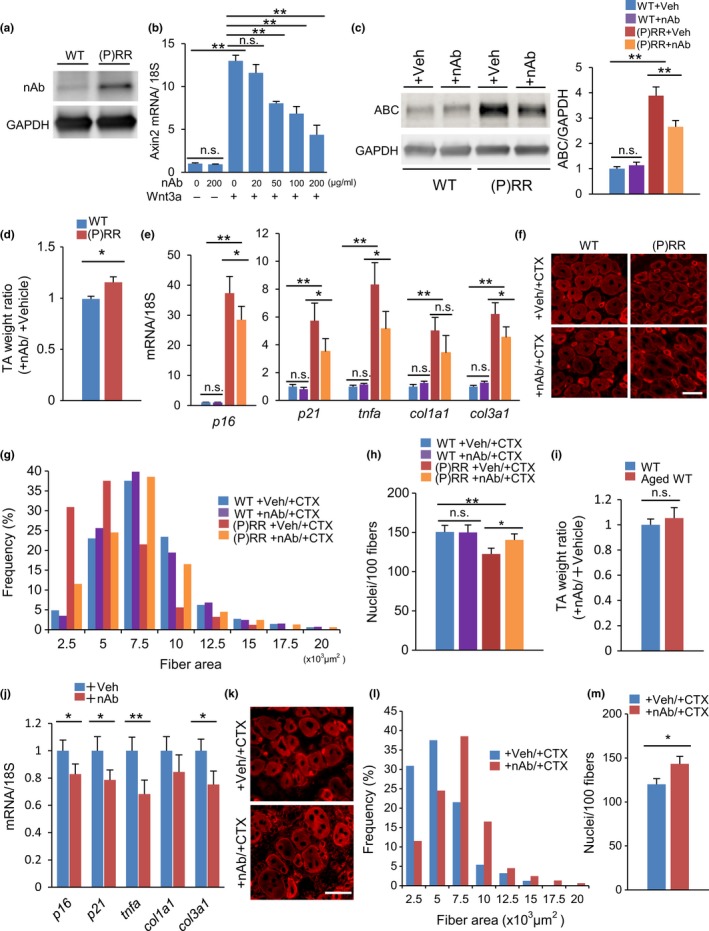
The anti‐(pro)renin receptor((P)RR) neutralizing antibody (nAb) restores muscle atrophy in (P)RR‐Tg mice and aged mice. (a) Validation of anti‐(P)RR nAb by Western blotting. Protein extract from (P)RR‐Tg mice was used as positive control. (b) Relative expression of *axin2* mRNA in Wnt3a‐stimulated C2C12 myoblasts treated with nAb. Expression levels were normalized to those of 18S rRNA and to those in myoblasts subjected to neither Wnt3a nor nAb treatment (*n* = 7). (c) Western blotting of active β‐catenin (ABC) expression in TA in WT and (P)RR‐Tg mice treated with nAb or vehicle (left) and quantification by densitometry (right) (*n* = 6). (d) Comparison of the weight ratio of nAb‐treated to vehicle‐treated TA muscle between WT and (P)RR‐Tg mice (*n* = 7). (e) Relative expression levels of *p16, p21, tnfa, col1a1*, and *col3a1* mRNA in muscles from WT and (P)RR‐Tg mice treated with nAb or vehicle (*n* = 7). (f) Immunofluorescence staining for embryonic MHC (eMHC) (red) in a cross section of muscle in WT and (P)RR‐Tg mice on day 5 after cardiotoxin (CTX) injection. Scale bars, 50 mm. (g) Cross‐sectional area (CSA) of eMHC‐positive myofibers (*n* = 7). (h) Number of nuclei per 100 myofibers (*n* = 7). (i) Comparison of the weight ratio of nAb‐treated to vehicle‐treated TA between young and aged mice (*n* = 7). (j) Relative expression levels of *p16, p21, tnfa, col1a1*, and *col3a1* mRNA in muscles from aged mice treated with nAb or vehicle (*n* = 6). (k) Immunofluorescent staining for eMHC (red) in a cross section of muscle in aged mice treated with nAb or vehicle on day 5 after CTX injection. Scale bars, 50 mm. (l) CSA of eMHC‐positive myofibers (*n* = 7). (m) Number of nuclei per 100 myofibers (*n* = 7). Data represent the mean ± *SEM*. *p < 0.05 and **p < 0.01; n.s., not significant, as determined by the Mann–Whitney *U* test (d,i,j,m) or ANOVA followed by the Bonferroni post hoc correction (b,c,e,h)

To clarify the importance of (P)RR binding to the Wnt receptor for activation of Wnt signaling, we used a neutralizing antibody (nAb) against (P)RR to inhibit its binding to the Wnt receptor. This nAb recognized (P)RR (Figure 5a) and suppressed the expression of *axin2* in C2C12 myoblasts stimulated with Wnt3a in a dose‐dependent manner (Figure 5b). The anti‐(P)RR nAb was injected into the atrophied muscle of (P)RR‐Tg mice, where it inhibited the activation of Wnt/β‐catenin signaling (Figure 5c), increased muscle weight 5 days after injection (Figure 5d), and reduced the expression of genes associated with inflammation, fibrosis, and senescence (Figure 5e). Next, to examine the effect of the anti‐(P)RR nAb on impaired regenerative capacity of myoblasts, the antibody was administered a day before inducing muscle injury by CTX. The nAb restored the muscle regenerative potential only in (P)RR‐Tg mice (Figure 5f–h). Finally, the nAb was administered to aged WT mice. The expression of muscle atrophy‐associated genes in aged WT mice was also suppressed by administration of the antibody, but the recovery of muscle mass was poor (Figure 5i,j). However, when muscle damage was widely induced at once by CTX, the nAb augmented muscle regenerative capacity even in aged WT mice (Figure 5k–m).

### 2.8 Hippo/YAP signaling is activated during skeletal muscle atrophy in (P)RR‐Tg mice and senile WT mice coordinately with the activation of Wnt/β‐catenin signaling

The Hippo/YAP signaling pathway is known to be cooperatively regulated by the activation of Wnt signaling via its release from the β‐catenin destruction complex (Azzolin et al., 2014). Therefore, we examined the involvement of YAP signaling in (P)RR‐induced activation of the Wnt/β‐catenin pathway and the pathogenesis of age‐related muscle atrophy.

**Figure 6:**
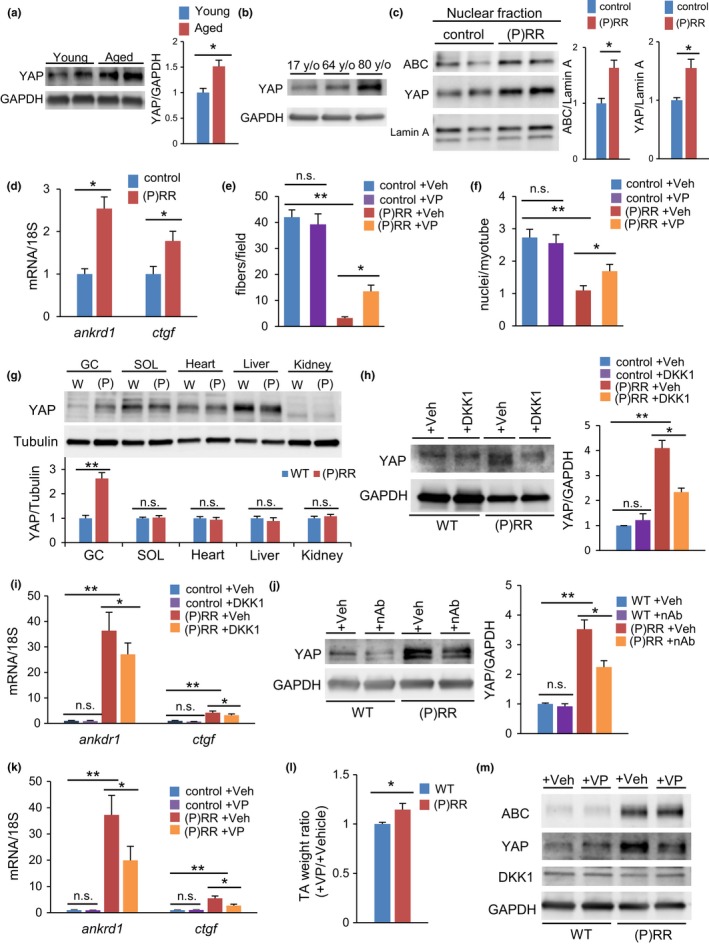
Yes‐associated protein (YAP) signaling is activated in sarcopenia coordinately with activation of Wnt signaling. (a) Western blotting of YAP in muscle from young and aged mice (left) and densitometry quantification (right) (*n* = 6). (b) Western blotting of YAP in muscles of 17‐, 64‐, and 80‐year‐old human volunteers. (c) Western blotting of active β‐catenin (ABC) and YAP using the nuclear fraction from (pro)renin receptor ((P)RR)‐expressing C2C12 myoblasts (left) and densitometry quantification (right) (*n* = 6). (d) Relative expression levels of *ankrd1* and *ctgf* mRNA in (P)RR‐expressing myoblasts (*n* = 6). (e) Number of MHC‐positive myotubes or (f) nuclei per differentiated myotube from (P)RR‐expressing myoblasts or controls treated with verteporfin or vehicle (*n* = 6). (g) Western blotting of YAP in gastrocnemius muscle, soleus muscle, heart, liver, and kidney of (P)RR‐Tg mice (left) and densitometry quantification (right) (*n* = 5). (h) Western blotting of YAP in muscles of WT and (P)RR‐Tg mice treated with DKK1 or vehicle (left) and densitometry quantification (right) (*n* = 6). (i) Relative expression levels of *ankrd1* and *ctgf* mRNA in muscles from WT and (P)RR‐Tg mice treated with DKK1 or vehicle (*n* = 7). (j) Western blotting of YAP in muscles of WT and (P)RR‐Tg mice treated with nAb or vehicle (left) and densitometry quantification (right) (*n* = 6). (k) Relative expression levels of *ankrd1* and *ctgf* mRNA in muscles from WT and (P)RR‐Tg mice treated with verteporfin or vehicle (*n* = 6). (l) Comparison of the weight ratio of verteporfin‐treated to vehicle‐treated TA in WT and (P)RR‐Tg mice (*n* = 7). (m) Western blotting of ABC, YAP, and DKK1 in muscles of WT and (P)RR‐Tg mice on day 5 after verteporfin or vehicle addition (*n* = 6). Data represent the mean ± *SEM*. *p < 0.05 and **p < 0.01; n.s., not significant, as determined by the Mann–Whitney *U* test (b,c,d,g,l) or ANOVA followed by the Bonferroni post hoc correction (e,f,h,i,j,k)

Yes‐associated protein expression increased in the atrophied muscles in senile mice and humans (Figure 6a,b). In C2C12 myoblasts, stimulation of Wnt3a enhanced YAP transcriptional activities in the TEAD‐binding element reporter (8×GTIIC) assay (Figure S5A), and upregulated downstream target genes of YAP, such as *ankrd1* and *ctgf* (Figure S5B), indicating that activation of Wnt signaling subsequently promoted YAP signaling. Moreover, in myoblasts stably harboring (P)RR, YAP was translocated into the nucleus in response to Wnt/β‐catenin activation (Figure 6c), stimulating expression of its target genes (Figure 6d). Wnt and YAP activities were enhanced in myotubes from (P)RR‐expressing C2C12 cells (Figure S5C). Treatment with verteporfin, a small molecule that inhibits the assembly of YAP/TEAD and its transcriptional activity, significantly restored myotube formation in (P)RR‐expressing myoblasts (Figure 6e,f).

Similar to the localization pattern of Wnt activation, YAP was more expressed in the skeletal muscle of (P)RR‐Tg mice than in WT mice, but other tissues did not follow suit (Figure 6g). Blockers of Wnt signaling, including DKK1 and anti‐(P)RR nAb, also suppressed YAP signaling in the muscle of (P)RR‐Tg mice (Figure 6h–j). Finally, administration of verteporfin improved muscle atrophy in (P)RR‐Tg mice via direct inhibition of YAP signaling (Figure 6k,l), without affecting ABC and endogenous DKK1 (Figure 6m), whereas in aged WT mice, administration of verteporfin suppressed the expression of sarcopenia‐related genes (Figure S5D).

## 3 DISCUSSION

The definition of sarcopenia remains unclear, even though its histological characteristics and senescence markers are well known (Larsson, 1983; Lexell, Taylor, & Sjostrom, 1988; Nilwik et al., 2013). Here, we defined sarcopenia if the following conditions were satisfied: (a) decrease in the number of muscle fibers (dominated by fast‐twitch fibers); (b) reduction in the size of each muscle fiber; (c) appearance of newly regenerated myocytes with central nuclei; (d) interstitial fibrosis and inflammation; (e) muscle weakness; and (f) increase in senescence markers, such as p16, p21 (Childs, Durik, Baker, & van Deursen, 2015), and SA‐β‐galactosidase activity (Dimri et al., 1995). The muscle of (P)RR‐Tg mice met all the above histological features of sarcopenia. Several genetic mouse models of muscle aging (Avin et al., 2014; Takeda, Hosokawa, & Higuchi, 1997) are presently available; however, their histological features are not fully compatible with sarcopenia. Aged WT mice, of course, are characterized by typical histological changes, but detecting them takes more than 100 weeks. The lack of an easy‐to‐use and reliable animal model for sarcopenia has slowed progress in this field. (P)RR‐Tg mice represent a particular sarcopenia model because they entail the activation of only one pathway, (P)RR‐Wnt‐YAP, responsible for eliciting sarcopenia. Nevertheless, they exhibit typical pathological tissue and functional decline, and hence, their application is expected to yield promising benefits in the study of aging‐related muscle atrophy.

(Pro)renin receptor is a multifunctional protein, involved in: (a) local activation of the renin–angiotensin system (RAS); (b) activation of mitogen‐activated protein kinase (MAPK) independently of (pro)renin; (c) regulation of lysosomal acidification as a subcomponent of V‐ATPase; and (d) activation of Wnt signaling. In humans (Onder et al., 2002) and mice (Yoshida et al., 2013), activation of RAS was shown to be involved in age‐related decline in muscle mass and function. Importantly, RAS inhibitors could suppress the progression of muscle atrophy. In the present study, the local concentration of angiotensin II in skeletal muscles of (P)RR‐Tg mice was within the normal range. As for lysosome acidification, ours and other groups have reported that the phenotypes observed after genetic ablation of (P)RR/Atp6ap2 were ascribed mainly to loss of V‐ATPase function (Kinouchi et al., 2010; Trepiccione et al., 2016), making it difficult to analyze other functions of (P)RR using a knockout model. To address this limitation, we developed here a “gain‐of‐function” model of (P)RR; however, even (P)RR overexpression failed to significantly affect lysosomal pH. Transgenic expression of (P)RR appeared to promote Wnt signaling, possibly by strikingly increasing the opportunities for (P)RR and Wnt receptors to come close to each other on the plasma membrane.

Muscle atrophy occurs when protein degradation exceeds protein synthesis; however, mTOR signaling was activated and UPP was suppressed in (P)RR‐Tg mice, suggesting that the muscle protein balance was shifted toward “synthesis” to compensate for the progression of muscle atrophy caused by another factor. In contrast, p62 was seen to accumulate and the conversion of LC‐3I to LC‐3II was attenuated, indicating that autophagic dysfunction could promote age‐related sarcopenia (Jiao & Demontis, 2017). In (P)RR‐Tg mice, autophagy dysfunction appeared first and was followed by the appearance of regenerated skeletal muscle fiber with central nuclei, as well as an increase in apoptotic cells and gradually more apparent muscle atrophy. Past reports suggested that an increase in (P)RR suppressed autophagy and induced apoptosis in age‐related muscle atrophy (Li & Siragy, 2015; Wohlgemuth, Seo, Marzetti, Lees, & Leeuwenburgh, 2010). Therefore, we believe that autophagy dysfunction induced by the activation of (P)RR‐Wnt‐YAP signaling triggers apoptotic cell death, which is followed by impaired muscle regeneration due to improper cell fusion of myoblasts.

A decrease in myocyte fusion results in smaller muscles with myofibers containing fewer nuclei (Horsley, Jansen, Mills, & Pavlath, 2003). (P)RR‐Tg mice exhibited myoblast fusion dysfunction during muscle regeneration following CTX injection. Past reports demonstrated that the activation of Wnt/β‐catenin signaling modified the muscle fusion process (Lacour et al., 2017; Rudolf et al., 2016) or drained the myogenic progenitor pool, followed by muscle atrophy (Rudolf et al., 2016). In the present study, the number of myogenin‐positive differentiating progenitors was higher in (P)RR‐Tg mice than in controls. As undifferentiated myoblasts are required for fusion into myotubes during postnatal muscle growth, we believe that aberrant activation of Wnt signaling by (P)RR promotes precocious differentiation of muscle progenitor cells, resulting in loss of cell fusion capacity and impaired muscle growth.

Although (P)RR‐Tg mice genetically expressed (P)RR in the entire body, muscle‐specific expression of (P)RR is pivotal for the sarcopenic phenotype. Here, muscle atrophy was induced by forced expression of (P)RR in skeletal muscle cell lines or, in vivo, through skeletal muscle‐specific expression *via* AAV. Activation of Wnt‐YAP signaling following increased (P)RR is tightly involved in the induction of skeletal muscle atrophy, but it remains unclear how (P)RR activates Wnt‐YAP signaling selectively in fast muscle. The Wnt receptor LRP6, a binding partner of (P)RR, has been reported to be predominantly expressed in fast muscle (Takeda et al., 2014). The expression pattern of LRP6 may contribute to fast muscle‐specific activation of the Wnt pathway in (P)RR‐Tg mice.

Wnt/β‐catenin and YAP signaling interact with each other, but the mechanism is not fully elucidated. In our results, inhibition of Wnt signaling by DKK1 attenuated the activity of YAP signaling, whereas inhibition of YAP signaling by verteporfin did not affect Wnt signaling activity, indicating that the latter occurred upstream of YAP in muscle cells. This finding is consistent with the results of a recent study, showing that Wnt signaling regulates the release of YAP into the nucleus *via* interaction with the β‐catenin destruction complex (Azzolin et al., 2014). In contrast, another study demonstrates that Wnt activates YAP through an alternative pathway, and so DKK1 could not suppress the activation of YAP (Park et al., 2015). In our experimental setting, inhibition of Wnt signaling by DKK1 reproducibly suppressed YAP activity in the atrophied muscles. The discrepancy between these different results can be attributed to the cell type, method, and administered dose of inhibitors.

Pharmacological inhibition of Wnt/β‐catenin/YAP signaling might have a therapeutic potential to attenuate age‐dependent muscle atrophy induced by (P)RR. For aged mice, it was difficult to assess the short‐term efficacy following single administration. However, in the muscle injury experiment using CTX, pharmacological blockade could restore the muscle regenerative capacity even in aged mice, indicating that inhibition of the (P)RR‐Wnt‐YAP pathway can be effective for the treatment of sarcopenia. Accordingly, repeated administration of anti‐(P)RR nAb or inhibitors of Wnt/β‐catenin signaling and/or YAP signaling, or using a viral vector that enables long‐term suppression of these signaling pathways might provide promising sarcopenia treatments, whose added benefit may be lifespan extension.

## CONFLICT OF INTEREST

None declared.
