# Supplementary material for: (Pro)renin receptor accelerates development of sarcopenia via activation of Wnt/YAP signaling axis
Source: Aging Cell. 2019 Jul 8;18(5):e12991. doi: 10.1111/acel.12991 (PMC6718617; doi:10.1111/acel.12991)

Supporting Information Figure S1

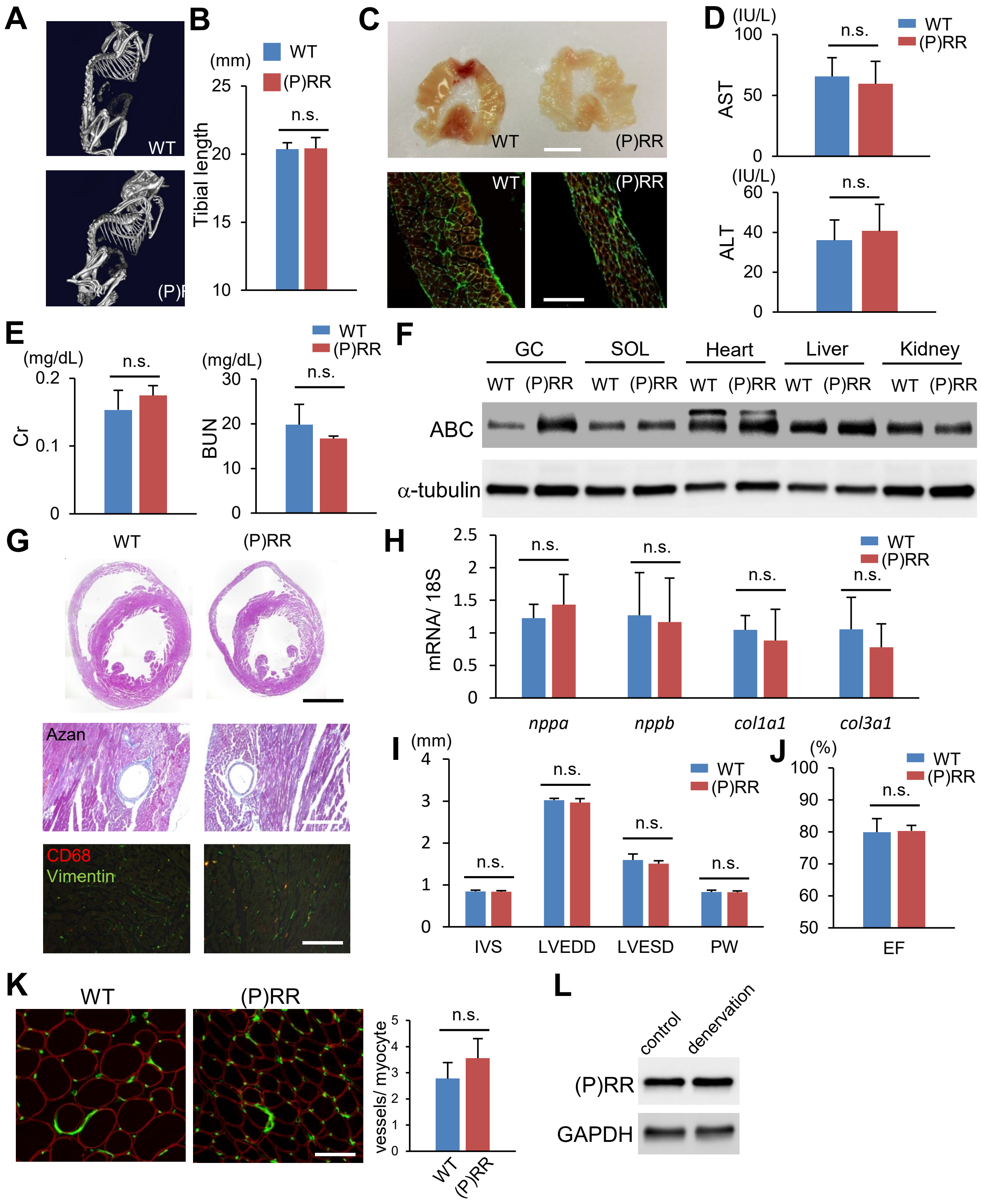

Supporting Information Figure S2

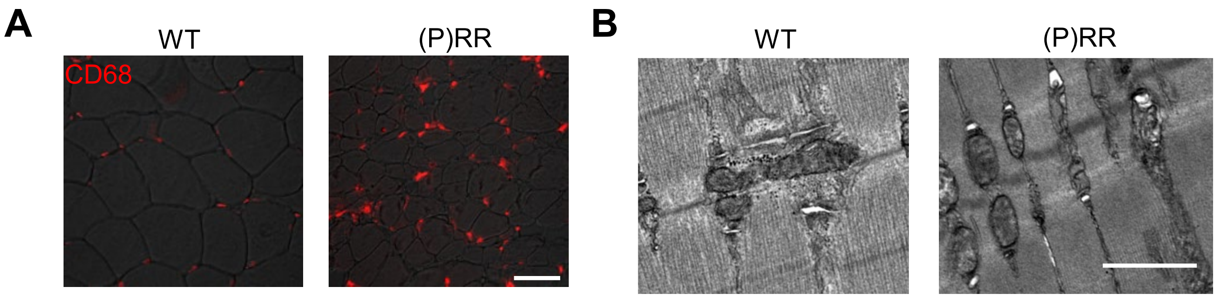

Supporting Information Figure S3

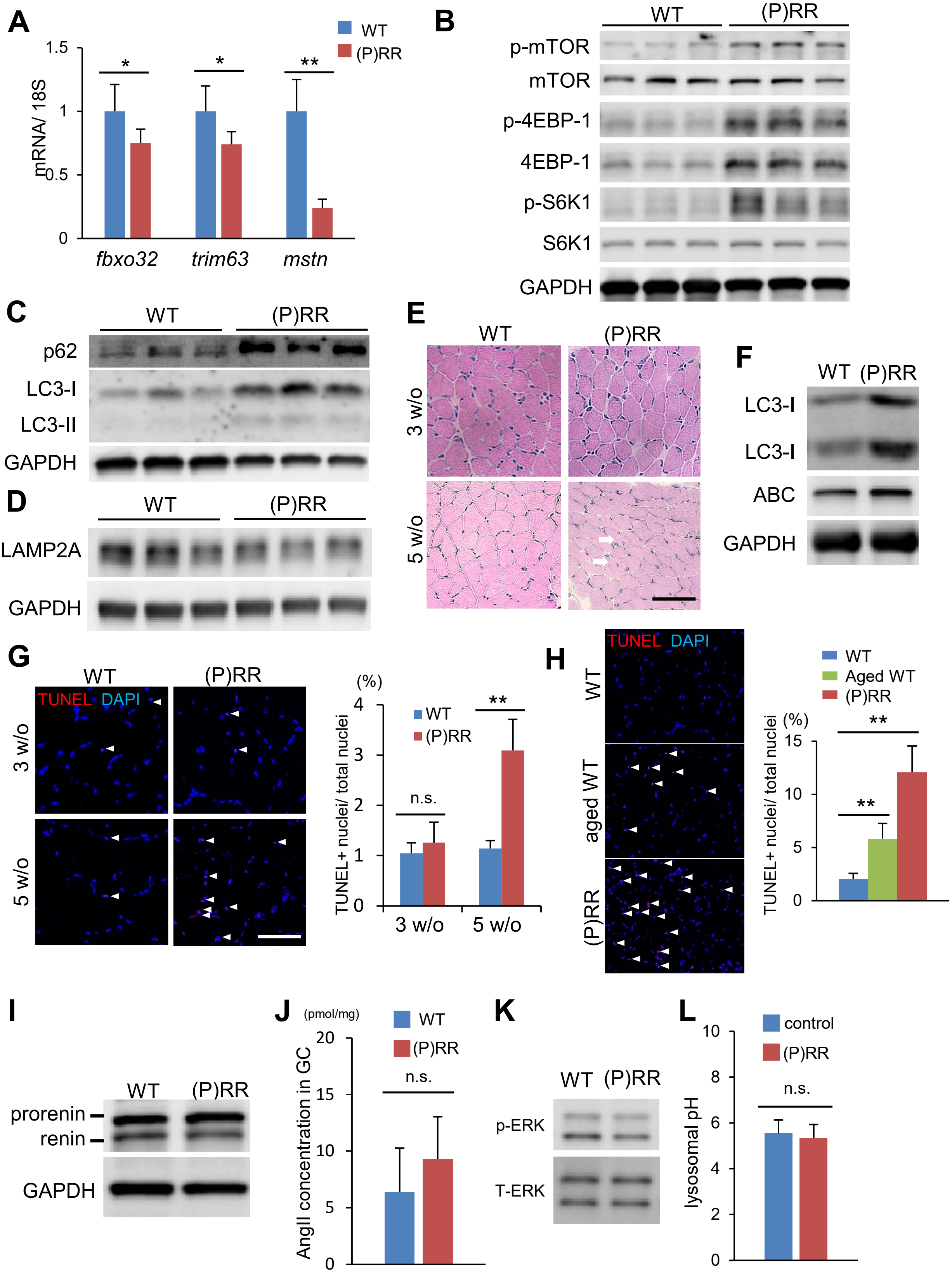

Supporting Information Figure S4

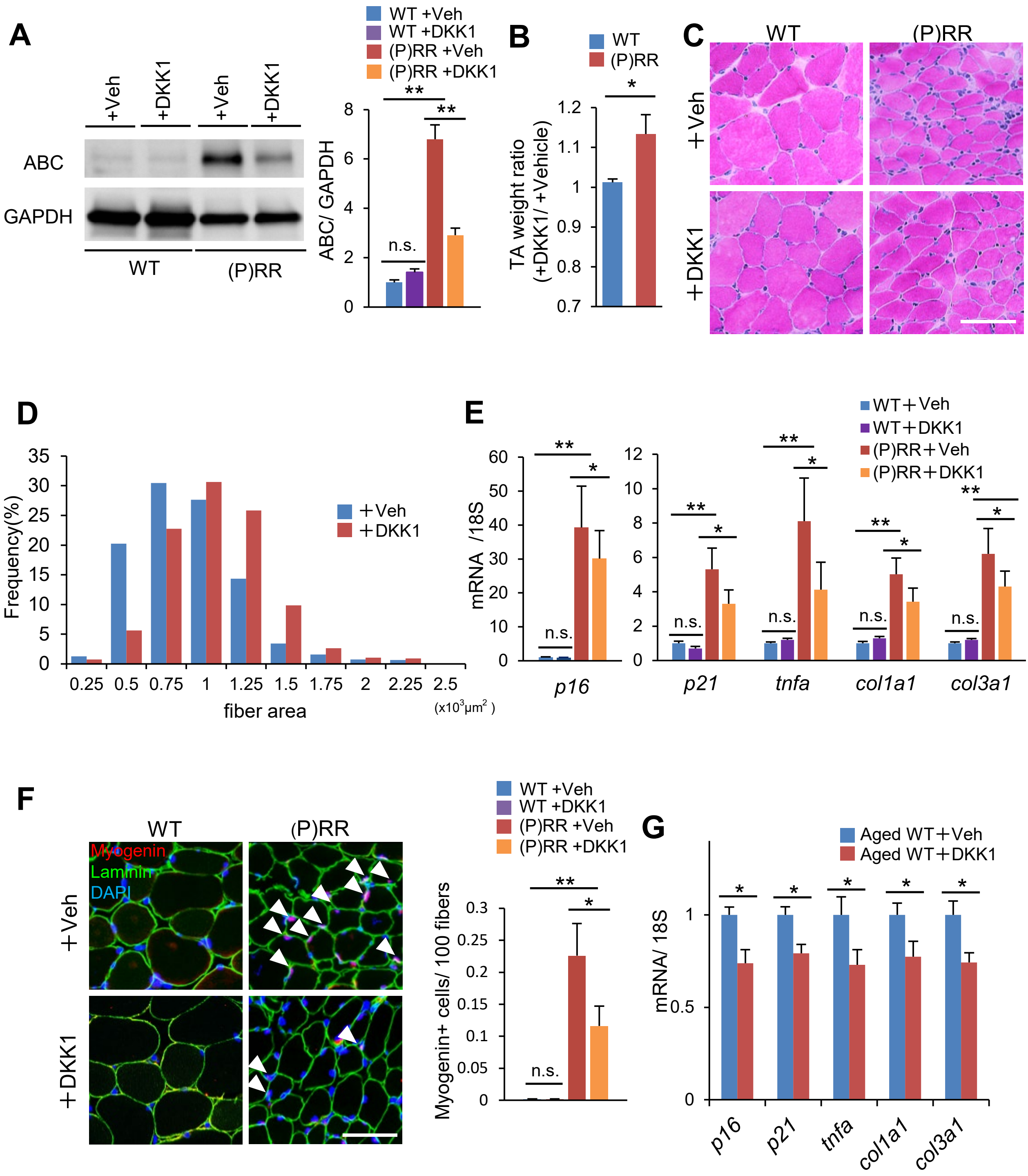

Supporting Information Figure S5

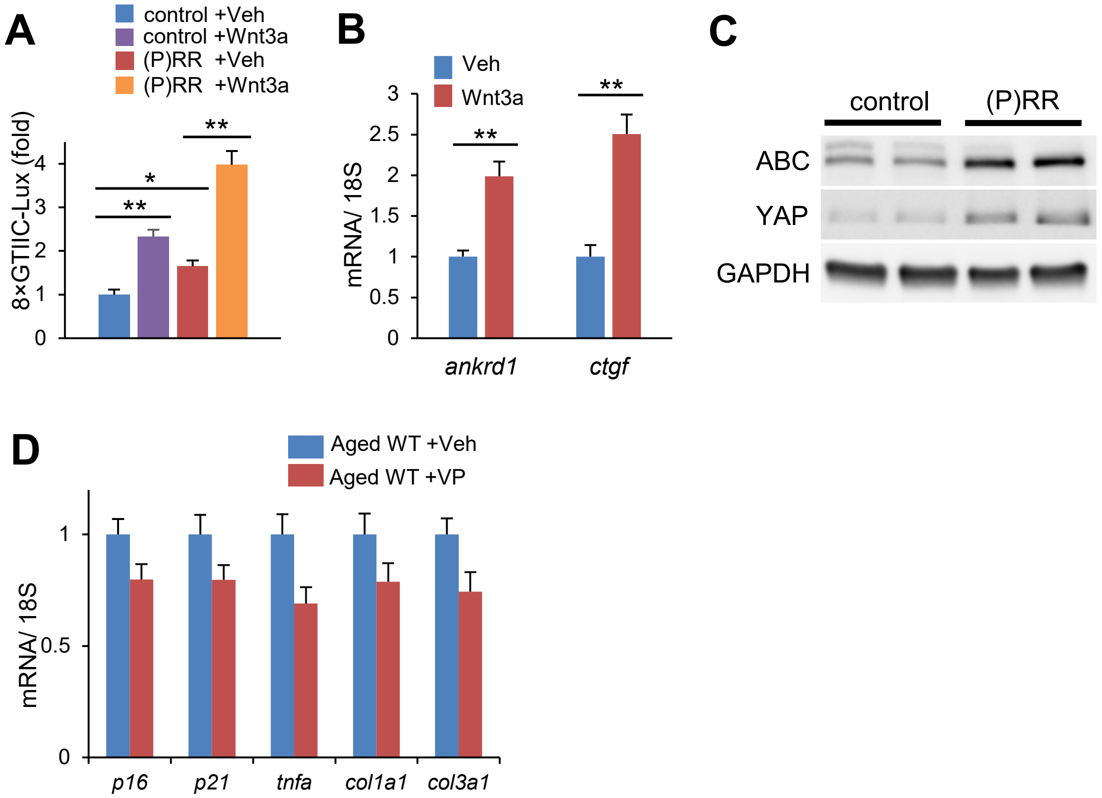

Supplement: Supplementary file 1 [file ACEL-18-e12991-s001.pdf]
