# Supplementary material for: (Pro)renin receptor accelerates development of sarcopenia via activation of Wnt/YAP signaling axis
Source: Aging Cell. 2019 Jul 8;18(5):e12991. doi: 10.1111/acel.12991 (PMC6718617; doi:10.1111/acel.12991)
Supplement: Supplementary file 2 [file ACEL-18-e12991-s002.pdf]

SUPPORTING INFORMATION

(Pro)renin receptor accelerates development of sarcopenia *via* activation of Wnt/ YAP signaling axis

Naohiro Yoshida[1,2], Jin Endo[2*], Kenichiro Kinouchi[3], Hiroki Kitakata[2], Hidenori Moriyama[2], Masaharu Kataoka[2], Tsunehisa Yamamoto[2], Kohsuke Shirakawa[2], Satoshi Morimoto[1], Akira Nishiyama[4], Akihiro Hashiguchi[5], Itsuro Higuchi[6], Keiichi Fukuda[2], Atsuhiro Ichihara[1] and Motoaki Sano[2]

ACKNOWLEDGMENTS

We thank K. Ito, Y. Shimizu, Y. Miyake, K. Yamashita, S. Goto, Y. Katsumata, T. Matsuhashi and N. Hayashiji for excellent technical assistance. We thank M. Kataoka, S. Yuasa and H. Takashima for valuable comments. This work was supported by a Grant-in-Aid for Scientific Research from the Japan Society for the Promotion of Science KAKENHI (18K07423, to J.E.) and (15H04825 and 15H01160, to M.S.), and a grant from the Takeda Science Foundation.

AUTHOR CONTRIBUTIONS

Conception and design: N.Y., J.E., K.K., A.I., and M.S.; development of methodology: N.Y., J.E., K.K. and I.H.; acquisition of data: N.Y., and J.E.; analysis and interpretation of data: H.K., H.M., S. I., T.Y., K.S., S.M., A.N. and A.H.; writing of manuscript: N.Y., and J.E.; study supervision: K.F., A.I., and M.S. All authors had the opportunity to comment on the manuscript.

MATERIALS AND METHODS

Animals

(P)RR-Tg mice were developed by pronuclear injection of a plasmid carrying mouse (P)RR under control of the cytomegalovirus enhancer chicken β-actin promoter and rabbit β-globin poly(A) signal (CAG) promoter. Non-Tg littermates were used as

controls. The mice used in these experiments were more than 10 generations back-crossed to C57BL/6 mice. (P)RR-Tg mice (C57BL/6 background) were bred in the Keio University animal facility.

Human subjects

Healthy adult volunteers were recruited to this study. Biopsies of triceps brachii muscle were performed.

Cell culture

C2C12 mouse myoblasts (ATCC, Manassas, VA, USA) were maintained in Dulbecco's modified Eagle's medium (DMEM) containing 20% fetal bovine serum. For myotube formation, the growth medium was replaced with a differentiation medium (DMEM with 2% horse serum).

Retrovirus production and generation of stable cell lines

Retrovirus was generated according to a previously reported protocol (Takahashi, Okita, Nakagawa, & Yamanaka, 2007). Briefly, pMXs-IRES GFP retroviral plasmid DNA encoding the (P)RR motif and GFP reporter cassette, was transfected into Plat-E packaging cells. Two days later, the conditioned medium of cultured Plat-E cells containing retroviruses was collected and filtered through 0.45-μm pore filters. Supernatant samples were applied immediately to C2C12 cell cultures. Polybrene (Sigma, St. Louis, MO, USA) was added at a final concentration of 8 mg/mL, and the supernatants were incubated with the cells for 12 h. After infection, the cells were placed in fresh growth medium and cultured as usual. Two days after infection, the cells were cultured in a 96-well plate at a limiting dilution. After one-week, individual clones expressing GFP picked from the plates of recombinant retrovirus-infected cells were transferred to 24-well microtiter plates and expanded to generate cell clones stably expressing mouse (P)RR.

TOPFlash assay

Luciferase reporter constructs were introduced into C2C12 cells by transient transfection using Lipofectamine® 2000 (Thermo Fisher Scientific, Waltham, MA, USA) and luciferase activity was determined with the ONE-Glo™ assay system (Promega, Madison, WI, USA). Wnt/β-catenin activity was determined using the TCF

reporter plasmid kit, including Super 8x TOPFlash and Super 8x FOPFlash plasmids (plasmid # 12456, Addgene, Cambridge, MA, USA).

Histological analysis and immunostaining

Frozen sections of GC or TA muscles were stained with hematoxylin and eosin (HE) to measure the cross-sectional area of the myofibers and count the number of regenerated myocytes having central nuclei, or with Sirius Red stain to detect fibrotic tissue.

Immunofluorescence experiments were carried out on 6-μm-thick frozen sections. The frozen sections were fixed with acetone for 20 min at -30 °C. The samples were incubated with 0.1% Triton X-100 for 5 min at room temperature, washed, and then incubated with the following primary antibodies: anti-laminin (1:1000; Sigma-Aldrich), anti-MHC type I (BA-F8, 1:50; DSHB, Iowa City, IA, USA), anti-MHC type IIA (SC-71, 1:600; DSHB), anti-MHC type IIX (6H1, 1:100; DSHB), anti-MHC type IIB (BF-F3, 1:100; DSHB), anti-myosin (MF20, 1:100; DSHB), anti-myogenin (M-225, 1:100; Santa Cruz Biotechnology, Dallas, TX, USA), anti-LC3 (EP1528Y, 1:100; Abcam, Cambridge, UK), anti-GFP antibody (598, 1:100; MBL, Nagoya, Japan), and anti-(P)RR antibody (ab40790, 1:100; Abcam). For the evaluation of vasculature, we used DyLight 488-conjugated Lycopersicon esculentum Lectin, (DL-1174, 1:100; Vector Laboratories, Burlingame, CA, USA) in place of immunostagining antibody. For staining of MHC types I, IIA, IIX, IIB, and myogenin, a mouse-on-mouse kit (Vector Laboratories, Burlingame, CA, USA) was used to block the endogenous mouse IgG. After overnight incubation at 4 °C, secondary antibodies conjugated with Alexa 488, Alexa 546, or Alexa 633 (Life Technologies, Carlsbad, CA, USA) were applied for 1 h at 4 °C. Nuclei were stained with 4',6-diamidino-2-phenylindole (DAPI; Molecular Probes, Eugene, OR, USA) in mounting medium, and the slides were visualized by confocal laser scanning microscopy (LSM510; Carl Zeiss, Jena, Germany) and fluorescence microscopy (BIOREVO BZ-9000; Keyence, Osaka, Japan).

For transmission electron microscopy, samples were prepared by standard procedures, sectioned using the RMC MT6000 ultramicrotome (Thermo Fisher Scientific), and visualized using a Hitachi H7500 electron microscope (Tokyo, Japan) and 2K × 2K Gatan CCD camera (Pleasanton, CA, USA).

Cellular senescence assay

C2C12 cells were fixed for 5 min in 3% formaldehyde and SA-β gal staining was performed using the senescence cells histochemical staining kit (Sigma-Aldrich).

Western blot analysis

Equal amounts of total protein isolated from skeletal muscle lysates were prepared in radioimmunoprecipitation assay buffer containing 50 mM Tris–HCl (pH 7.6), 150 mM NaCl, 1% Nonidet-P40, 0.5% sodium deoxycholate, and 0.1% sodium dodecyl sulfate (SDS), and supplemented with 1 mM dithiothreitol (DTT), 100 nM MG132, protease inhibitor cocktail (Nacalai Tesque, Kyoto, Japan), and phosphatase inhibitor cocktail. Nuclear proteins were isolated from cultured cells by the NE-PER™ nuclear and cytoplasmic extraction kit (Thermo Fisher Scientific) according to the manufacturer's instructions. For western blot analysis, equal amounts of total protein (15–20 µg) from skeletal muscle lysates were subjected to SDS-polyacrylamide gel electrophoresis. The primary antibodies used were as follows: anti-(P)RR (R&D Systems, Minneapolis, MN, USA), anti-active-β-catenin (Millipore, Billerica, MA, USA), anti-γH2AX and anti-renin antibody (Abcam), anti-Lamp2 (DSHB), anti-phospho-p44/42 MAPK, anti-p44/42 MAPK, anti-β-actin, anti-α-tubulin, anti-glyceraldehyde 3-phosphate dehydrogenase (GAPDH), anti-phospho-AKT (Thr308), anti-AKT (pan), anti-phospho-4E-BP1 (Thr37/46), anti-4E-BP1, anti-phospho-mTOR (Ser2448), anti-mTOR, anti-phospho-p70 S6 kinase (Thr389), anti-p70 S6 kinase, anti-LRP6, and anti-YAP (all Cell Signaling Technology, Danvers, MA, USA). Protein bands were visualized using horseradish peroxidase-conjugated secondary antibodies and enhanced chemiluminescence (Amersham Biosciences, Piscataway, NJ, USA) on a LAS-3000 luminoimager (Fujifilm Techno Products,, Kanagawa, Japan). Protein bands were quantified using ImageJ (NIH, Bethesda, MD, USA).

RNA extraction and real-time PCR

Total RNA was extracted by acid guanidinium thiocyanate-phenol-chloroform extraction with TRIZOL reagent. Reverse transcription was performed using the Prime Script™ RT reagent kit (Takara, Otsu, Japan), in accordance with the manufacturer’s protocol. Quantitative real-time PCR was performed using the ABI Prism 7700 sequence detection system (Applied Biosystems, Waltham, MA, USA). Predesigned gene-specific primer and probe sets (TaqMan Gene Expression Assays) were purchased from Applied Biosystems. The 18S ribosomal RNA was amplified and used as an internal control.

Handgrip strength test

Five training sessions were performed, during which the mice were held, facing the bar of a grip strength meter (GPM-100; Melquest, Toyama, Japan), while the forearm was

gently restrained by the experimenter. When the unrestrained forepaw was brought into contact with the bar of the grip strength meter, the mouse grasped the bar. Then, the mouse was gently pulled away from the device. The grip strength meter measured the maximal force applied before the animal released the bar.

Skeletal muscle injury

CTX (*Naja mossambica mossambica*, 10 μM; Sigma-Aldrich) diluted in 50 μL of phosphate-buffered saline (PBS) was injected into the TA muscle of BL6/J mice using a 29-gauge needle and 1-mL syringe. The needle was inserted deep into the TA muscle longitudinally toward the knee and CTX was injected along the length of the muscle. Mice in the control group were injected with 50 μL of PBS. Mice in both the treatment and control groups were sacrificed at various time points after CTX injection.

TUNEL assay

For in situ detection of apoptotic cells, TUNEL assay was performed using the In Situ Cell Death Detection Kit, TMR red (Sigma-Aldrich) according to the manufacture's protocol.

Intramuscular injection of DKK-1, verteporfin, and anti-(P)RR neutralizing antibody

The reagents diluted in PBS were mixed with a thermo-reversible gelation polymer (Mebiol® Gel, Advanced BioMatrix, San Diego, CA, USA) and injected into the TA muscle of (P)RR-Tg mice or WT mice using a 29-gauge needle. The final concentrations of recombinant mouse DKK1 (R&D Systems), verteporfin (Chemscene, Monmouth Junction, NJ, USA), and anti-(P)RR neutralizing antibody (a kind gift from Dr. A. Nishiyama, Kagawa University, Japan) were adjusted to 500 ng/50 μL, 100 μg/50 μL, and 67 μg/50 μL, respectively.

Production and Purification of rAAV

Adeno-associated virus (AAV) serotype 2 vector was generated by a triple transfection of HEK-293T cell line with pAAV2 insert containing mouse Atp6ap2 under the control of CMV, followed by IRES, GFP gene as a reporter, pAAV-RC2 containing rep and cap genes of AAV serotype 2, and pHelper encoding the adenovirus helper functions. Viral vectors were extracted from the transfected cells by AAVpro Extraction solution (Takara) and purified by AAVpro Purification kit (all serotypes) (Takara) according to the manufacture's protocol.

.

Intramuscular Injection of AAV

The AAV solution was mixed with a thermo-reversible gelation polymer. Right TAs in 8-week-old WT mice were injected with 1.0 x 10^{11} vg/TA in 20 μL of the gelatin polymer mixed with either AAV2 expressing mAtp6ap2-IRES-GFP or AAV2 expressing only IRES-GFP as control, and same dose of the gelatin polymer with PBS was injected in the contralateral TA.

Echocardiography and hemodynamic measurements.

Mice were anesthetized by 1.5% isoflurane inhalation, and then anchored to a positioning platform in the supine position. Short-axis echocardiographic and Doppler echocardiographic measurements were made using the Vevo 660 system (Visual Sonics) with a 600 series real-time microvisualization scanhead probe. The interventricular septum (IVS), posterior wall (PW), LV internal end-systolic and end-diastolic diameters (LVESD and LVEDD, respectively) were measured using the leading-edge convention adopted by the American Society of Echocardiography. Ejection fraction (EF) was calculated according to the formula: EF (%) = ([LVEDV – LVESV]/LVEDV) × 100. Heart rate did not differ significantly among the groups during the echocardiographic assessments.

Study approval

The animal study conformed to NIH guidelines (*Guide for the Care and Use of Laboratory Animals*. National Academies Press. 1996.) and was approved by the IACUC of the Keio University School of Medicine (reference number 15061). All aspects of the study involving humans conformed to the principles outlined in the Declaration of Helsinki, and all experiments were performed after obtaining written informed consent. The human study was approved by the Human Research Ethics Committee of Kagoshima University (reference number 28-129).

Statistical Analysis

Values are presented as means ± standard error of the mean (SEM). Comparisons between two groups were carried out using the Mann-Whitney U test, whereas comparisons among multiple groups were performed using ANOVA followed by Bonferroni post-hoc correction. A P-value < 0.05 was considered statistically significant. Statistical analyses were performed using SPSS 22.0 for Windows (SPSS Inc., Chicago, IL, USA).

REFERENCES

Takahashi, K., Okita, K., Nakagawa, M., & Yamanaka, S. (2007). Induction of pluripotent stem cells from fibroblast cultures. *Nat Protoc*, *2*(12), 3081-3089. doi:10.1038/nprot.2007.418

FIGURES LEGENDS

Supplemental Figure S1 (P)RR-Tg mouse exhibit no apparent phenotype in skeleton, liver, kidney or heart. (**A**) 3D-CT images of whole skeleton in WT (top) and (P)RR Tg mice (bottom). (**B**) Tibial length in WT and (P)RR-Tg mice (*n*=9). (**C**) Representative low-magnification views of the diaphragm of WT (top, left) and (P)RR-Tg mice (top, right). Scale bars, 10mm. Immunostaining for laminin (green) and MHC IIB (red) of cross sections of the diaphragms in WT (bottom, left) and (P)RR-Tg mice (bottom, right). Scale bars, 200mm. (**D**) Liver and (**E**) kidney blood test (AST, ALT, Cr and BUN) of WT and (P)RR-Tg mice(*n*=6). (**F**) Total proteins were extracted from the skeletal muscles (GC, SOL), heart, liver and kidney of WT and (P)RR-Tg mice and the expression of active b-catenin (ABC) protein was analyzed by Western-blotting (left). (**G**) Representative low-magnification views of the histological sections with Azan staining (macro) of heart in WT and (P)RR-Tg mice (top). Scale bars, 2mm. Histological sections with Azan staining (micro) of heart in WT and (P)RR-Tg mice (middle). Scale bars, 200mm. Immunostaining of cross-sections of heart in WT and (P)RR-Tg mice for CD68 (red) and Vimentin (green) (bottom). Scale bars, 200mm. (**H**) Relative expression levels of *nppa, nppb, col1a1 and col3a1* mRNA in hearts from WT and (P)RR-Tg mice. Expression levels were normalized to those of 18S ribosomal RNA (*n*=6). (**I**) IVS, LVEDD, LVESD, PW and (**J**) EF in WT and (P)RR Tg mice measured by echocardiography. (n=6), (**K**) The immunostaining for laminin (red) and the vascular staining by using lectin (green) (left) and the number of vessels around each myofiber in a cross-section of TA muscle in WT and (P)RR Tg mice. Scale bars, 100mm. (**L**) Western blot analysis of (P)RR expression in the muscle of the mice after sham operation or denervation. Data represent the mean ± SEM. n.s., not significant, as determined by the Mann-Whitney U test.

Supplemental Figure S2 Juvenile (P)RR-Tg mouse exhibit no apparent phenotype in the skeletal muscle. (**A**) Immunostaining for CD68 (red) of cross

sections of GC in WT and (P)RR-Tg mice. Scale bar, 50mm. (**B**) Electron microscopic images of GC in WT and (P)RR-Tg mice. Scale bar, 1mm.

Supplemental Figure S3 Autophagy dysfunction is an initial trigger of sarcopenia in (P)RR-Tg mice. (**A**) Relative expression levels of *fbxo32* (atrogin-1), *trim63* (Murf1) and *mstn* (myostatin) mRNA in GC of WT and (P)RR-Tg mice. Expression levels were normalized to those of 18S ribosomal RNA and then normalized with respect to those in the GC of WT mice (*n*=6). (**B**) Total proteins were extracted from the GC of WT and (P)RR-Tg mice and the phosphorylation of mTOR, 4EBP-1 and S6K1 were analyzed by Western-blotting (*n*=5). (**C, D**) Total proteins were extracted from the GC of WT and (P)RR-Tg mice and the protein expression of p62, LC3 (C) and Lamp2A(D) were analyzed by Western-blotting (*n*=5). (**E**) Histological sections with hematoxylin and eosin (HE) staining of GC from 3 w/o WT, 5 w/o WT, 3 w/o (P)RR-Tg, and 5 w/o (P)RR-Tg mice. Scale bar, 100 mm. (**F**) Western blot analysis of LC3 and active β-catenin (ABC) in total protein extracts from the TA muscle of WT and (P)RR-Tg mice both at 3 weeks after birth. GAPDH was used as internal control for total protein. (**G**) TUNEL staining in a section of GC muscle from 3 w/o WT, 5 w/o WT, 3 w/o (P)RR-Tg, and 5 w/o (P)RR-Tg mice. Nuclei were labeled with DAPI (blue). Scale bar, 50 mm. (left). The ratio of the TUNEL-positive nuclei number to total nuclei number (right). (**H**) TUNEL staining in a section of WT, aged WT and (P)RR Tg mice. Nuclei were labeled with DAPI (blue). Scale bar, 100 mm. (left). The ratio of the TUNEL-positive nuclei number to total nuclei number (right). (**I**) Western blot analysis of renin and prorenin in total protein extracts from the GC muscle of WT and (P)RR-Tg mice. (**J**) The tissue concentration of endogenous Angiotensin II in GC of WT and (P)RR-Tg mice (*n*=5). (K)Total proteins were extracted from the GC of WT and (P)RR-Tg mice and the phosphorylation of ERK was analyzed by Western-blotting (*n*=6). (**L**) Comparison of the lysosomal pH between control C2C12 cells and (P)RR-expressing C2C12 cells by using pHrodo™ Green Dextran (*n*=9). Data represent the mean ± SEM. **P* < 0.05, ***P* < 0.01; n.s., not significant, as determined by the Mann-Whitney U test.

Supplemental Figure S4 Inhibition of Wnt/b-catenin signaling by administration of DKK1 attenuates sarcopenia-like muscle atrophy in (P)RR-Tg mice. (**A**) Western blot analysis of active b-catenin (ABC) expression in total protein extracts from the TA muscle of WT and (P)RR-Tg mice treated with DKK1 or vehicle (left) and quantification by densitometry (right). GAPDH was used as internal control for total protein (*n*=6). (**B**) Comparison of the weight ratio of DKK1:vehicle-treated TA muscle

between WT and (P)RR-Tg mice (*n*=7). (**C**) Hematoxylin and eosin (HE) staining of the TA muscle five days after administration of DKK1 or vehicle in WT and (P)RR-Tg mice. Scale bars, 100 mm. (**D**) Cross-sectional area of individual muscle fibers in the TA muscle of (P)RR-Tg mice treated with DKK1 or vehicle (n=7, N=450–500 per group). (**E**) Relative expression levels of *p16, p21, tnfa, col1a1, and col3a1* mRNA in TA muscles from WT and (P)RR-Tg mice three days after administration of DKK1 or vehicle. Expression levels were normalized to those of 18S ribosomal RNA and then to those in the TA muscle of WT mice treated with vehicle (*n*=6). (**F**) Immunofluorescent staining for myogenin (red) and laminin (green) in cross-sections of TA muscles five days after administration of DKK1 or vehicle in WT and (P)RR-Tg mice. Nuclei were labeled with DAPI (blue). White arrowheads correspond to myogenin-positive nuclei. Scale bars, 150 mm (left). Numbers of myogenin-positive myocytes in WT and PRR-Tg mice (*n*=6, N=450–500 per group) (right). **(G)** Relative expression levels of *p16, p21, tnfa, col1a1, and col3a1* mRNA in TA muscles from aged WT mice three days after administration of DKK1 or vehicle. Expression levels were normalized to those of 18S ribosomal RNA and then to those in the TA muscle of WT mice treated with vehicle (*n*=6). Data represent the mean ± SEM. *$P < 0.05$, **$P < 0.01$; n.s., not significant, as determined by the Mann-Whitney U test (B, G) or ANOVA followed by Bonferroni *post-hoc* correction (A, E, F).

Supplemental Figure S5 YAP signaling is activated by stimulation of Wnt3a in C2C12 myoblasts. (**A**) YAP-responsive luciferase reporter, 8xGTIIC-Luciferase, activities of control or (P)RR-expressing C2C12 myoblasts in the presence or absence of Wnt3a (*n*=6). (**B**) Relative expression levels of the downstream target genes of YAP (*ankrd1* and *ctgf*) in C2C12 cells in the presence or absence of Wnt3a stimulation. Expression levels were normalized to those of 18S ribosomal RNA and then normalized with respect to those in vehicle group (*n*=6). (**C**) Western blot analysis of active β-catenin (ABC) and YAP in total protein extracts from the control or (P)RR-expressing C2C12 myotube. GAPDH was used as internal control for total protein. **(D)** Relative expression levels of *p16, p21, tnfa, col1a1, and col3a1* mRNA in TA muscles from aged WT mice three days after administration of verteporfin or vehicle. Expression levels were normalized to those of 18S ribosomal RNA and then to those in the TA muscle of WT mice treated with vehicle (*n*=6). Data represent the mean ± SEM. **$P < 0.01$; n.s., not significant, as determined by ANOVA followed by Bonferroni *post-hoc* correction (A) or the Mann-Whitney U test (B, D).
